# Characterization of the thermophilic xylanase Fsa02490Xyn from the hyperthermophile *Fervidibacter sacchari* belonging to glycoside hydrolase family 10

**DOI:** 10.1002/2211-5463.70072

**Published:** 2025-08-29

**Authors:** Nicole Torosian, Jonathan K. Covington, Allison M. Cook, Nancy O. Nou, Marike Palmer, Ritesh Mewalal, Miranda Harmon‐Smith, Ian K. Blaby, Jan‐Fang Cheng, Matthias Hess, Brian P. Hedlund

**Affiliations:** ^1^ School of Life Sciences University of Nevada, Las Vegas NV USA; ^2^ Department of Microbiology University of Manitoba Winnipeg Canada; ^3^ US Department of Energy Joint Genome Institute Lawrence Berkeley National Laboratory CA USA; ^4^ Department of Animal Science, College of Agricultural and Environmental Sciences University of California, Davis CA USA; ^5^ Nevada Institute of Personalized Medicine University of Nevada, Las Vegas NV USA

**Keywords:** *Armatimonadota*, GH10, glycoside hydrolase, hyperthermophile, xylanase

## Abstract

*Fervidibacter sacchari* is an aerobic hyperthermophile belonging to the phylum *Armatimonadota* that degrades a variety of polysaccharides. Its genome encodes 117 enzymes with one or more annotated glycoside hydrolase (GH) domain, but the roles of these putative GHs in polysaccharide catabolism are poorly defined. Here, we describe one *F. sacchari* enzyme encoding a GH10 domain, Fsa02490Xyn, that was previously shown to be active on *Miscanthus*, oat β‐glucan, and beech‐wood xylan, with optimal activity at 90–100 °C. We show that Fsa02490Xyn is also active on birch‐wood xylan and gellan gum. The pH range on beech‐wood xylan was 4.5 to 9.5 (pH_opt_ 7.0–8.0). Fsa024940Xyn had a *K*_m_ of 2.375 mm, *V*_max_ of 1250 μm·min^−1^, and *k*_cat_/*K*_m_ of 1.259 × 10^4^ s^−1^·m ^−1^ when using a *para*‐nitrophenyl‐𝛽‐xylobioside assay. A phylogenetic analysis of GH10 family enzymes revealed a large clade of enzymes from diverse members of the class *Fervidibacteria*, including Fsa02490Xyn and a second enzyme from *F. sacchari*, with apparent horizontal gene transfer within *Fervidibacteria* and between *Fervidibacteria* and thermophilic *Bacillota*. This study establishes Fsa02490Xyn as a hyperthermophilic GH10 enzyme with endo‐β‐1,4‐xylanase activity and identifies a large clade of homologous GH10 enzymes within the class *Fervidibacteria*.

**Impact statement**

The depolymerization of xylan at high temperatures is important because this process limits the degradation of polysaccharides in nature and the synthesis of biofuels from plant wastes. Our study is also important because *F. sacchari* is one of only a few cultivated members of the *Armatimonadota*, which are polysaccharide‐degradation specialists.

## Graphical abstract

*Fervidibacter sacchari* is a member of the bacterial phylum *Armatimonadota*, only a few of which have been cultivated. It is a hyperthermophilic polysaccharide degradation specialist. We show that a secreted enzyme, Fsa02490Xyn, is an endoxylanase belonging to glycoside hydrolase family 10. It is active as a dimer with optimal activity at 90–100 °C.

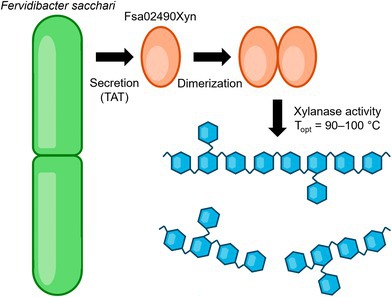

**Abbreviations**
AFEX: ammonia fiber expansion
CBM: carbohydrate‐binding module
CSM: computed structure model
DNS: 3,5‐dinitrosalicylic acid
GH: glycoside hydrolase
GH10: glycoside hydrolase family 10
HGT: horizontal gene transfer
HSD: Tukey's honestly significant difference
IPTG: Isopropyl‐β‐thiogalactoside
LB: lysogeny broth
PAE: predicted aligned error
*p*NPX2: *para‐*nitrophenyl‐β‐xylobioside
SDS/PAGE: sodium dodecyl sulfate‐polyacrylamide gel electrophoresis
TIM: triose‐phosphate isomerase
UPLC‐MRM‐MS: ultra‐performance liquid chromatography‐multiple reaction monitoring‐mass spectrometry

Hyperthermophilic bacteria and archaea have optimal growth temperatures that exceed 80 °C [1]. Natural habitats for hyperthermophiles include physicochemically diverse terrestrial and marine hydrothermal systems at both the surface and in the subsurface [2]. Because these environments are not conducive to plant growth, they are typically depauperate in organic matter, including polysaccharides. Despite this, some thermophiles and hyperthermophiles can catabolize polysaccharides through fermentation or by coupling the oxidation of component sugars to the reduction of terminal electron acceptors through aerobic or anaerobic respiration.

Regardless of the downstream energy‐coupling reactions, polysaccharide catabolism is initiated by glycoside hydrolase (GH) enzymes that depolymerize polysaccharides into oligo‐ or monosaccharides, which are then transported into the cytoplasm and catabolized. Members of several different GH families can degrade xylan, and those with optimal activity on xylan are classified as xylanases. Xylan is the major component of hemicellulose, which is a major component of plant cell walls and the second most abundant polysaccharide on Earth [3]. It is composed of a linear chain of β‐(1,4)‐d‐xylosyl residues α‐1,2‐ or α‐1,3‐linked arabinofuranose residues and a variety of substitutions such as glucuronic acid, ferulic acid, and *para*‐coumaric acid [3]. Xylanases from a variety of thermophiles have been characterized, including those that contain GH family 10 (GH10) domains derived from members of the phyla *Bacillota* (e.g., *Caldicellulosiruptor* [4, 5, 6], *Clostridium* [7, 8, 9], *Geobacillus* [10, 11, 12], and *Thermoanaerobacterium* [13, 14, 15]); *Bacteroidota* (*Rhodothermus*) [16, 17, 18]; *Dictyoglomota* (*Dictyoglomus*) [19, 20]; *Ignavibacteriota* (*Melioribacter*) [21]; and *Thermotogota* (*Thermotoga*) [22, 23, 24].

Recently, a polysaccharide‐degrading hyperthermophile, *Fervidibacter sacchari*, was isolated from cellulolytic enrichments originating from Great Boiling Spring, Nevada [25], by using interpretations from a single‐cell genome [26] and a metagenome‐assembled genome (MAG) [27] to guide cultivation. *F. sacchari* is unusual among polysaccharide‐degrading hyperthermophiles based on its aerobic metabolism and membership to the phylum *Armatimonadota* and class *Fervidibacteria*, both of which are resistant to cultivation but composed of known or predicted polysaccharide degraders, based on cultivation [28, 29, 30] and/or a high number of annotated GH genes [25]. *F. sacchari* grows optimally at 80 °C on various hemicelluloses, glucans, and plant biomass substrates, and its genome encodes 117 annotated GH enzymes. Of the 117 predicted GH enzymes encoded in the *F. sacchari* genome, Fsa02490Xyn is one of two with annotated GH10 domains. A previous study [25] showed Fsa02490Xyn to be thermophilic (*T*_opt_ 90–100 °C), with activity on beech‐wood xylan, oat β‐glucan, and *Miscanthus*. That study also showed that Fsa02490Xyn is among the most highly expressed GH genes during growth on eight individual polysaccharides, with maximal expression when *F. sacchari* was grown with locust bean gum or xyloglucan as carbon sources. Here, we heterologously expressed, purified, and further characterized Fsa02490Xyn to better understand its biochemical properties and potential role in polysaccharide catabolism.

## Materials and methods

### DNA synthesis, heterologous expression, purification, and computed structure modeling

To determine the GH family of Fsa02490Xyn, its amino acid sequence was submitted for annotation by the dbCAN2 meta server [31]. The presence of a signal peptide was also assessed by submitting the Fsa02490Xyn amino acid sequence to SignalP [32]. To confirm the presence of two GH10‐conserved glutamic acid residues, a MAFFT‐DASH multiple sequence alignment of Fsa02490Xyn along with four other GH10 amino acid sequences was conducted and visualized in jalview v. 2.11.2.6 [33]. The Fsa02490Xyn gene was codon‐optimized with an *E. coli* codon frequency table [34] and synthesized (Twist BioScience, South San Francisco, CA, USA) inclusive of 30mer flanking linkers enabling assembly into the BamHI site of the pET21b vector with NEBuilder HiFi Assembly (Novagen, Madison, WI, USA; New England Biolabs, Ipswich, MA, USA). The resulting construct contained a 6 × His tag, a GB1 solubility tag, and a TEV protease cleavage site at the N terminus, was sequence verified on the Pacific Biosciences Sequel IIe platform and transformed into *E. coli* T7 Express cells (New England Biolabs) for expression.

To express Fsa02490Xyn, *E. coli* cells containing its gene were initially grown on lysogeny broth (LB) (10 g·L^−1^ tryptone, 5 g·L^−1^ yeast extract, 5 g·L^−1^ NaCl) agar (15 g·L^−1^) plates with 100 μg·mL^−1^ ampicillin, then transferred to LB broth with 100 μg·mL^−1^ ampicillin, and incubated at 37 °C with shaking at 125 r.p.m. until reaching an OD_600_ = 0.6–0.8. Isopropyl‐β‐thiogalactoside (IPTG) was added to a concentration of 0.5 mm, and the culture continued incubating overnight at 37 °C with shaking at 80 r.p.m. The cells were centrifuged for 2 min at 16 100 ***g***, then resuspended in lysis buffer (50 mm Tris/HCl, 100 mm NaCl, pH 7.0), and lysed by sonication for 40 s with 40% cycle and level 4 output using a Branson 450 Sonifier (Branson Ultrasonics, Brookfield, CT, USA). To separate the soluble fraction, the resultant lysate was clarified by centrifugation for 2 min 16 100 ***g***.

Fsa02490Xyn was further purified using immobilized metal affinity chromatography with HisPur™ Ni‐NTA Spin Columns (Thermo Scientific, Rockford, IL, USA). The columns were pre‐equilibrated using 20 mm Na_2_HPO_4_ buffer with 300 mm NaCl and 10 mm imidazole, and the soluble fraction containing Fsa02490Xyn was loaded. The columns were washed with 20 mm Na_2_HPO_4_ buffer containing 300 mm NaCl and 25 mm imidazole, and Fsa02490Xyn was eluted using 20 mm Na_2_HPO_4_ buffer with 300 mm NaCl and 250 mm imidazole. Imidazole was removed from purified Fsa02490Xyn via buffer exchange into lysis buffer using Pierce™ Protein Concentrators with a 10 K molecular weight cutoff (Thermo Scientific, Rockford, IL, USA). Fsa02490Xyn concentration was quantified using a Qubit® Protein and Protein Broad Range Assay Kit (Thermo Fisher Scientific, Waltham, MA, USA) with a Qubit® 3.0 Fluorometer (Thermo Fisher Scientific, Waltham, MA, USA). For experiments using the native and mature form of Fsa02490Xyn, 15 μg of the purified enzyme was mixed in 1× TEV protease reaction buffer (50 μL final volume). Then, 10 units of TEV Protease (New England Biolabs) were added, and the mixture was incubated at 30 °C for 1 h to catalyze the cleavage of the recombinant protein at the TEV protease site.

The expression, purity, and molecular weights of recombinant and TEV protease‐treated Fsa02490Xyn were assessed with 7.5% SDS/PAGE using a PageRuler™ Pre‐stained Protein Ladder (Thermo Scientific, Waltham, MA, USA). The oligomerization capability of Fsa02490Xyn was assessed using 7.5% native PAGE with a PageRuler™ Plus Pre‐stained Protein Ladder (Thermo Scientific, Vilnius, Lithuania) with and without TEV protease pretreatment.

To evaluate the structure of Fsa02490Xyn, computed structure models (CSMs) of Fsa02490Xyn were created using alphafold 2 via the colabfold platform integrated with chimerax v. 1.5 using default settings [35, 36]. Six Fsa02490Xyn CSMs were made: (a) native and mature Fsa02490Xyn (signal peptide removed); (b) non‐native Fsa02490Xyn containing an N‐terminal 6 × His tag, GB1 solubility tag, and a TEV protease site; (c) homodimeric native and mature Fsa02490Xyn (signal peptides removed); (d) homotrimeric native and mature Fsa02490Xyn (signal peptides removed); and (e) homotetrameric native and mature Fsa02490Xyn (signal peptides removed). The generated CSMs were visualized and edited in chimerax v. 1.5, and the potential for Fsa02490Xyn to homooligomerize was assessed by evaluating the predicted alignment error (PAE) plots of the multimeric CSMs.

### Enzyme substrate assays

Analysis of potential substrates was conducted in pH‐optimized lysis buffer (50 mm Tris/HCl, 100 mm NaCl, pH 7.5) at 80 °C. An empty vector control was run in parallel as a negative control; empty vector controls consisted of protein preparations derived from *E. coli* T7 Express cells containing pET‐21b‐GB1 that were grown and treated with IPTG in parallel to cells expressing Fsa02490Xyn. To determine its enzymatic activity, Fsa02490Xyn was tested with the following potential substrates, which were previously shown to be used by *F. sacchari* as sole carbon sources and electron donors [25]: β‐glucan from oat (Megazyme, Bray, Ireland), chondroitin sulfate (Alfa Aesar, Ward Hill, MA, USA), colloidal chitin [37] (Beantown Chemicals, Hudson, NH, USA), fucoidan from brown algae (BestVite, Sun Valley, CA, USA), galactan from lupin (Megazyme), gellan gum (Serva, Heidelberg, Germany), glycogen from oysters (TCI, American Fork, UT, USA), karaya gum (Sigma, Cream Ridge, NJ, USA), locust bean gum (Spectrum, New Brunswick, NJ, USA), starch from potatoes (J.T. Baker, Phillipsburg, NJ, USA), xanthan gum (Sigma), xylan from beech wood (Megazyme), xylan from birch wood (Sigma), xyloglucan from tamarind (CarboMer), as well as AFEX‐pretreated corn stover, *Miscanthus*, and sugarcane bagasse (Dupont, Wilmington, DE, USA). These substrates (0.5% w/v in sterile ddH_2_O, final concentration) with added Fsa02490Xyn (40.4 μg·mL^−1^, final concentration) or the same volume of an empty vector control in microplate wells (final volume 40 μL), then sealed and incubated overnight at 80 °C alongside a beaker of water to prevent evaporation. To quantify reducing sugars released from the reactions, 3,5‐dinitrosalicylic acid (DNS) solution (0.25 g DNS, 75 g sodium potassium tartrate, 50 mL of 2 m NaOH, brought to 250 mL with ultrapure water) was added to each well (final volume 200 μL), then the microplate was wrapped in foil and incubated for 20 min at 100 °C. The absorbance of each well at 570 nm was measured using a SpectraMax® Plus 384 Absorbance Plate Reader. Substrates seemingly degraded by Fsa02490Xyn were retested in triplicate and compared to the empty vector control using unpaired one‐way *t*‐tests (*P* < 0.05). A parallel experiment was conducted where TEV protease‐pretreated Fsa02490Xyn was used. DNS standard curves were generated using 0, 5, 10, 15, 20, and 25 mm glucose.

To assess the β‐xylosidase activity of Fsa02490Xyn, *p*NPX2 was tested. *p*NPX2 (20 μg·mL^−1^, final concentration) was mixed 1 : 1 with Fsa02490Xyn (40.4 μg·mL^−1^, final concentration) and incubated for 30 min at 80 °C. Na_2_HPO_3_ buffer (2% w/v, pH 12.0) was used to stop the reaction, and the absorbances of each well at 400 nm were measured. A standard curve was generated using 0, 6, 12, 18, 24, and 30 μg·mL^−1^ *para*‐nitrophenol.

### Biochemical characterization and kinetic parameters

The pH range and optimum were determined by first buffer‐exchanging Fsa02490Xyn into 50 mm buffers at a range of pHs in 0.5 intervals: citrate (pH 3.0–5.0), 2‐morpholine‐4‐ethanesulfonic acid (5.5–6.5), 2‐amino‐2‐(hydroxymethyl)propane‐1,3‐diol (7.0–9.0), 2‐(cyclohexylamino)ethane‐1‐sulfonic acid (9.5–10.0), and 3‐(cyclohexylamino)propane‐1‐sulfonic acid (10.5–11.0), all containing 100 mm NaCl. Buffer‐exchanged Fsa02490Xyn (40.4 μg·mL^−1^, final concentration) was mixed 1 : 1 vol/vol with xylan (0.5% w/v, final concentration) from beech wood dissolved in the same buffers, then incubated at 90 °C for 1 h. Thermostability was assessed by preincubating Fsa02490Xyn (40.4 μg·mL^−1^, final concentration) at 90, or 100 °C for 1 h in pH 7.5 buffer, then incubating at 90 °C with 0.5% xylan from beech wood. The temperature range and optimum were determined previously [25] by incubating a different preparation of Fsa02490Xyn with xylan from beech wood (0.5% w/v, final concentration) at 4, 20, 30, 40, 50, 60, 70, 80, 90, 100, 110, or 120 °C. All assays were performed in triplicate along with triplicate empty vector controls, and the resultant reducing sugars were quantified using the DNS assay. The temperature and pH ranges were determined by comparing to the empty vector control with an unpaired one‐way *t*‐test (*P* < 0.05). The temperature and pH optima and thermostability were determined using one‐way analysis of variance (ANOVA) with *post hoc* Tukey's honestly significant difference (HSD) tests (*P* < 0.05).

### Kinetic parameters

The Michaelis–Menten kinetic parameters *k*_cat_ and *k*_cat_/*K*_M_ of Fsa02490Xyn were determined by combining Fsa02490Xyn (0.697 μm) with *p*NPX2 at a range of concentrations (0.625, 1.25, 2.5, 5, 10, and 20 mm) and incubating for 11 min under optimal conditions (pH 7.5, 90 °C). The reactions were terminated and the amount of *para*‐nitrophenol released from the reactions were assessed as before. A Lineweaver–Burk plot was generated to calculate *K*_m_, *V*_max_, *k*_cat_, and *k*_cat_/*K*_m_.

### Metabolomics following Fsa02490Xyn digestion of beech‐wood xylan

To assess the hydrolytic products of beech‐wood xylan digestion by Fsa02490Xyn, an aliquot of lysate from a 1 h enzymatic digest (pH 7.5, 90 °C) was analyzed by UPLC‐MRM/MS at The Metabolomics Innovation Centre (Edmonton, AB, Canada). The digest was centrifuged to remove solids and frozen at −20 °C until shipment on dry ice for analysis. The thawed digest was lyophilized, dissolved in 80% methanol, vortexed, sonicated, and then centrifuged. About 60 μL of supernatant was mixed with 20 μL of an internal standard solution containing ^13^C‐labeled sugars (fructose, galactose, glucose, mannose, ribose, and xylose) in 50% methanol, 30 μL 3‐nitrophenylhydrazine‐HCl (100 mm), and 30 μL 1‐ethyl‐3‐(3‐dimethylaminopropyl)carbodiimide‐HCl‐3% pyridine solution (100 mm), then incubated for 60 min at 50 °C. About 10 μL of the resultant solution was added to a pentafluorophenyl column to run UPLC‐MRM/MS on an Agilent 1290 UHPLC coupled to an Agilent 6495B QQQ mass spectrometer. Standards of 14 compounds at eight dilutions were simultaneously prepared and included: 4‐O‐methyl‐d‐glucuronic acid, fructose, fucose, galactose, glucose, glucuronic acid, lactose, maltose, mannose, rhamnose, ribose, ribulose, xylose, and xylulose.

### Phylogenetic analysis of GH10

To analyze the phylogeny of GH10 enzymes, all amino acid sequences of characterized GH10s available in the CAZy database [38] were retrieved from the National Center for Biotechnology Information (NCBI; https://www.ncbi.nlm.nih.gov) using the Batch Entrez site (https://www.ncbi.nlm.nih.gov/sites/batchentrez). Fsa02490Xyn (NCBI locus_tag Q2T83_02490; GenBank protein ID WKU16701.1), along with all other GH10 homologs from high‐quality *Fervidibacteria* genomes (28 total), were added, then duplicate sequences and sequences of fewer than 50 amino acids were removed using bioedit v. 7.2.5 [39], resulting in 548 total amino acid sequences. The mafft online server v. 7 was used to generate a multiple sequence alignment with the mafft‐dash structural alignment algorithm. The alignment was subjected to a true maximum likelihood analysis using iqtree v. 2.3.6 [40] using ultrafast bootstrapping (UFBoot) and Shimodaira–Hasegawa approximate likelihood ratio test (SH‐aLRT) branch support values [41] from 1000 replicates each. The resultant phylogenies were analyzed with the interactive tree of life v. 6 website (https://itol.embl.de) and edited in inkscape v. 1.2.2.

## Results and Discussion

### Annotation, computed structure modeling, and multimeric structure of Fsa02490Xyn

Fsa02490Xyn was previously predicted to encode a GH10 domain based on HMMER (e‐value 2.1e‐57), dbCAN‐sub (e‐value 6.1e‐117), and DIAMOND (e‐value 9.8e‐131). Pfam PF00331 (glycosyl hydrolase family 10) was also identified in the Fsa02490Xyn gene, further supporting the annotation of a GH10 domain. No other domains were annotated within Fsa02490Xyn. SignalP predicted a TAT N‐terminal signal sequence (likelihood 0.83322), suggesting that Fsa02490Xyn is secreted as a folded protein. The mature Fsa02490Xyn is 430 amino acids, which is typical among GH10 enzymes [42, 43]. A multiple sequence alignment of Fsa02490Xyn with four characterized thermophilic GH10 enzymes confirmed the presence of two conserved glutamic acid residues, Glu201 and Glu305 (Glu166 and Glu270 in the mature form, respectively) (Fig. S1).

To assess the structure of Fsa02490Xyn, a CSM was created using alphafold 2 within the colabfold platform. The CSM of the native protein had low PAE scores (0–5) with the crystal structure of AcXyn10A (PDB ID: 8B73), an endo‐β‐1,4‐xylanase from *Acetivibrio clariflavus* DSMZ 19732 (40.81% identity, BlastP e‐value = 6e‐98), suggesting a highly accurate CSM (Fig. S2A,B). In this model, an apparent active site was visible within a (β/α)8 triose‐phosphate isomerase (TIM) barrel based on the juxtaposition of the two conserved glutamic acid residues, Glu166 and Glu270, facing the hollow core formed by the eight alternating β‐sheets and α‐helices of the (β/α)8 TIM structure (Fig. S2C). Based on structure–function studies of other GH10 enzymes [14, 44, 45], Glu166 and Glu270 likely serve as the catalytic nucleophile and catalytic proton donor. The C‐terminal 58 amino acids of the CSM form a separate β‐barrel‐like structure resembling a β‐sandwich‐like structure but composed of only seven β‐sheets and with one pair of juxtaposed β‐sheets being parallel instead of antiparallel. Some GH10 enzymes have known or annotated carbohydrate‐binding module (CBM) domains, including an annotated C‐terminal CBM2 domain in the *Cellulomonas fimi* ATCC 484 Xyn10A to which Fsa02490Xyn was aligned (Fig. S1). However, no CBMs or jelly rolls were annotated in Fsa02490Xyn previously and InterProScan also failed to annotate this domain. A CSM was created for the full recombinant protein that includes an N‐terminal GB1 solubility tag, 6 × His tag, and TEV protease site, and was truncated to remove the predicted N‐terminal TAT signal sequence (Fig. S2D). This CSM had low PAE scores (0–5), suggesting correct folding of the recombinant protein used for the majority of experiments, described below. CSMs were also generated with native and mature Fsa02490Xyn homodimers, homotrimers, and homotetramers, yet these showed high PAE scores between monomers (dimers 15–30; trimers and tetramers 25–30), suggesting a monomeric structure for Fsa02490Xyn.

**Fig. 1:**
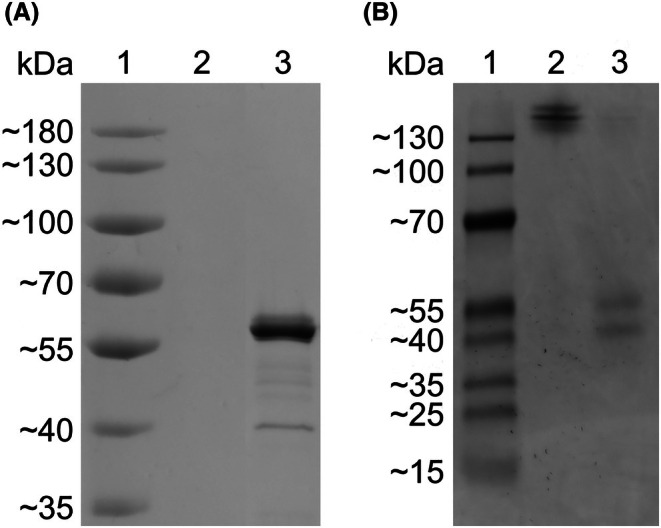
SDS/PAGE and native PAGE of purified Fsa02490Xyn. (A) Purified proteins were visualized on a 7.5% polyacrylamide SDS/PAGE gel. Lane 1: PageRuler™ Pre‐stained Protein Ladder (Thermo Scientific, Waltham, MA, USA); 2: empty vector control; 3: recombinant Fsa02490Xyn with N‐terminal 6 × His tag, GB1 solubility tag, and TEV protease site (0.808 μg). The expected molecular mass of the recombinant protein is ~ 53 KDa. (B) Purified proteins were visualized on a 7.5% polyacrylamide native PAGE gel. Lane 1: PageRuler™ Plus Pre‐stained Protein Ladder (Thermo Scientific, Vilnius, Lithuania); 2: recombinant Fsa02490Xyn with N‐terminal 6 × His tag, GB1 solubility tag, and TEV protease site intact. 3: recombinant Fsa02490Xyn with N‐terminal 6 × His tag, GB1 solubility tag, and TEV protease site removed following treatment with TEV protease (0.808 µg). Coomassie brilliant blue was used to stain both gels. The original image was spliced to remove empty lanes.

After expression of Fsa02490Xyn in *E. coli* T7 Express cells and purification by immobilized metal affinity chromatography, the purity and mobility of the recombinant protein was determined by using both sodium dodecyl sulfate‐polyacrylamide gel electrophoresis (SDS/PAGE) and native PAGE. SDS/PAGE revealed a single protein with an estimated size of approximately 58 kDa (Fig. 1A). The recombinant protein was slightly larger than the expected size of the native protein (48 kDa) due to the combined effect of removing the TAT signal sequence (3 kDa) and the addition of the GB1 solubility tag (6 kDa), 6 × His tag (1 kDa), and TEV protease site (1 kDa). Some minor polypeptides smaller than 50 kDa were also visualized by SDS/PAGE. Since these minor products were not in an empty vector control protein preparation derived from *E. coli* T7 Express cells containing pET‐21b‐GB1, they were probably degradation products of Fsa02490Xyn.

Native PAGE of the full recombinant enzyme, including the N‐terminal GB1 tag, 6 × His tag, and TEV protease site, revealed a double band above 130 kDa (Fig. 1B), initially suggesting that the recombinant Fsa02490Xyn formed a multimeric structure consisting of at least two Fsa02490Xyn monomers. However, after digestion with TEV protease, we observed a band at the expected size of the monomer ~ 48 kDa and a second band at ~ 42 kDa. We suggest the smaller bands on these gels may also be proteolytic products. This result agrees with the predicted monomeric structure of Fsa02490Xyn based on PAE values, described above. Out of 58 GH10 structures available from CAZy, 48 are monomeric, nine are homodimeric, one is heterodimeric, and none are trimeric or larger. Thus, the monomeric structure of Fsa02490Xyn is typical of the GH10 family.

### Substrate specificity of Fsa02490Xyn

**Fig. 2:**
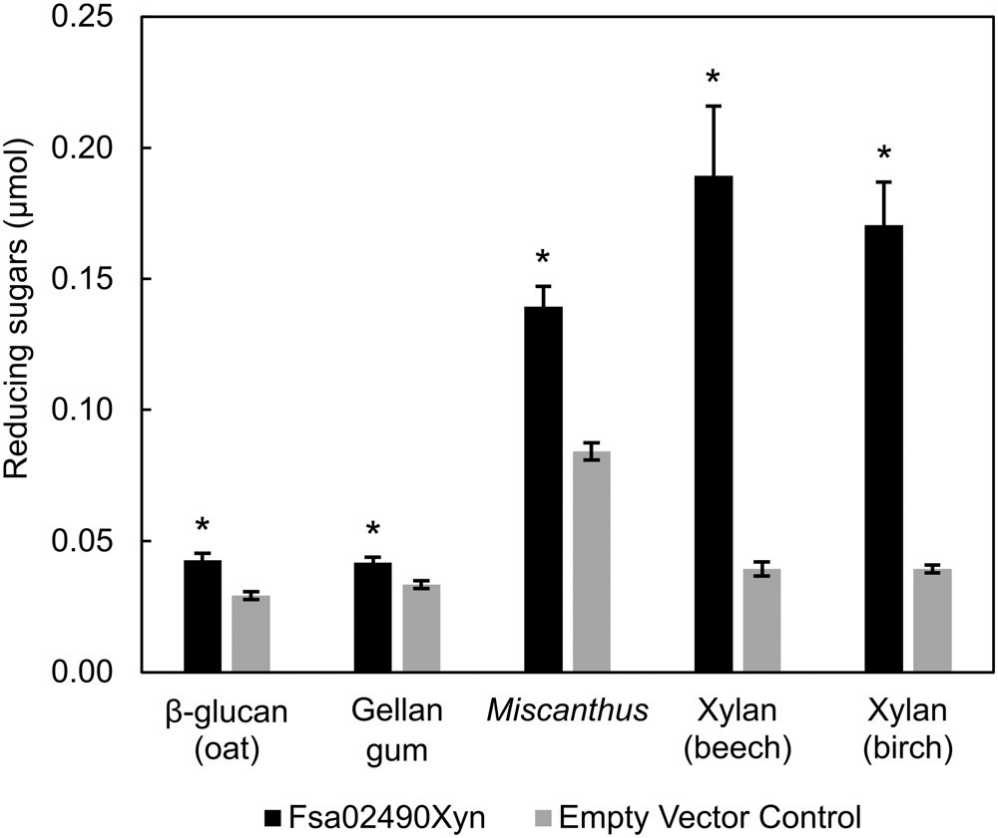
Substrate specificity. Fsa02490Xyn is active on β‐glucan from oat, gellan gum, AFEX‐pretreated *Miscanthus*, xylan from beech wood, and xylan from birch wood compared to the empty vector control after an overnight incubation (*n* = 3, *P < 0.005 via an unpaired one‐way *t*‐test). Error bars indicate standard deviation. Assays were done with 40.4 μg·mL^−1^ (final concentration) of Fsa02490Xyn and 0.5% substrate (final concentration) at pH 7.5 at 80 °C (prior to temperature optimization).

The recombinant Fsa02490Xyn was screened against 14 purified polysaccharides previously shown to be used by *F. sacchari* PD1^T^ as sole carbon sources and electron donors [25] and ammonia fiber expansion (AFEX)‐pretreated corn stover, *Miscanthus*, and sugarcane bagasse. Using this screen, we confirmed previously reported activity of Fsa02490Xyn on beech‐wood xylan and *Miscanthus* and report for the first‐time activity on xylan from birch wood and gellan gum (*P* < 0.05 versus an empty vector control, unpaired *t*‐test) (Fig. 2), with TEV protease pretreatment having no effect on β‐glucan degradation (Fig. S3). Activity against both xylans and glucans indicates some activity on polysaccharides with both five‐ and six‐carbon sugar backbones; however, the > 5‐fold higher yield of reducing sugars from both forms of xylan compared with β‐glucan and gellan gum indicates a preference for polysaccharides containing five‐carbon sugar monomers. As lignocellulose contains polysaccharides with both five‐carbon sugar backbones (hemicellulose) and six‐carbon sugar backbones (cellulose), intermediate activity against *Miscanthus* is consistent with this interpretation. Since xylan is composed of a backbone of β‐(1,4)‐D‐linked xylose residues [3], this result is consistent with the annotations described above and the optimal activity of most characterized GH10 enzymes as endo‐β‐1,4‐xylanases [43, 46]. This function was supported by Fsa02490Xyn activity on the colorimetric substrate *para‐*nitrophenyl‐β‐xylobioside (*p*NPX2) (Fig. S4) and with the absence of xylose or any other sugar monomers detected by ultra‐performance liquid chromatography‐multiple reaction monitoring‐mass spectrometry (UPLC‐MRM‐MS) following Fsa02490Xyn digestion of beech‐wood xylan. The latter result suggests that Fsa02490Xyn cleaves glycosidic bonds within oligo‐ and polysaccharides with chain length ≥ 4, liberating oligo‐ and polysaccharides but not monosaccharides. The low activity of Fsa02490Xyn on β‐glucan and gellan gum was likely due to low aryl cellobiosidase activity previously described for some GH10 endo‐β‐1,4‐xylanases [47].

### Optimization of Fsa02490Xyn activity, thermostability, and kinetics

**Fig. 3:**
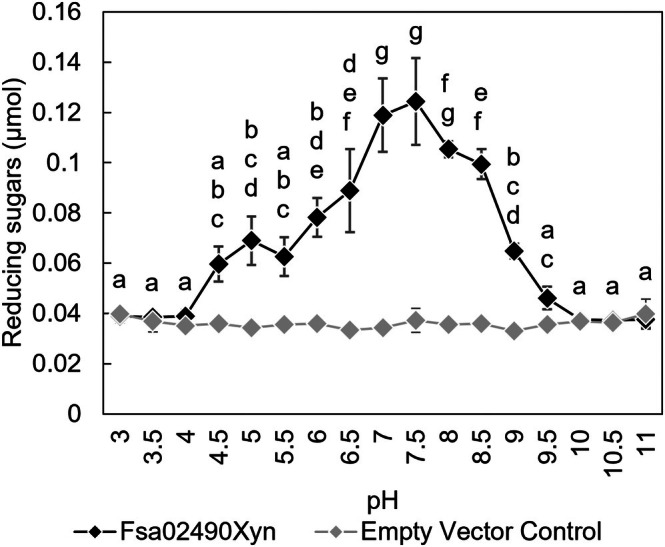
pH range and optimum of Fsa02490Xyn. Fsa02490Xyn was optimally active at pH 7–8 on beech‐wood xylan. Treatments with a shared letter are not significantly different (*n* = 3, *P* < 0.05 via a one‐way ANOVA with *post hoc* Tukey's HSD). Error bars indicate standard deviation. Fsa02490Xyn was active at a pH range of 4.5 to 9.5 when compared to the empty vector control (*P* < 0.05 via unpaired *t*‐tests). Assays were done with 40.4 μg·mL^−1^ (final concentration) of Fsa02490Xyn and 0.5% substrate (final concentration) at 90 °C.

The temperature optimum of Fsa02490Xyn on beech‐wood xylan was reported previously as 90–100 °C [25]. We report here that enzyme activity on beech‐wood xylan occurred at all temperatures tested (4–120 °C) (Fig. S5). The low but significant activity at 120 °C makes Fsa02490Xyn among the most thermophilic known GH10 enzymes, along with XynA from *Thermotoga neapolitana* [22] and an enzyme designated Xyn10K from an uncultivated thermophile [48], discussed below. The pH range for activity on beech‐wood xylan was 4.5 to 9.5, with an optimum of 7.0–8.0 (Fig. 3). These conditions are similar to those that permit growth of *F. sacchari*, which are 65–87.5 °C and pH 6.5 to 8.6 [25], and the physicochemical conditions of Great Boiling Spring, which is the only known habitat of *F. sacchari* [25, 27, 49]. Other thermophilic GH10 enzymes have been characterized, including those from thermophilic members of the phyla *Bacillota* (e.g., *Caldicellulosiruptor* [4, 5, 6], *Clostridium* [7, 8, 9], *Geobacillus* [10, 11, 12], and *Thermoanaerobacterium* [13, 14, 15]); *Bacteroidota* (*Rhodothermus*) [16, 17, 18]; *Dictyoglomota* (*Dictyoglomus*) [19, 20]; *Ignavibacteriota* (*Melioribacter*) [21]; and *Thermotogota* (*Thermotoga*) [22, 23, 24] (Table 1).

**Table 1:** Properties of selected thermophilic GH10 enzymes that have been characterized.

Enzyme	Organism^a^	Phylum	Activity	*T*_opt_ (°C)	References
Fsa02490Xyn	*Fervidibacter sacchari* PD1^T^	*Armatimonadota*	Endoxylanase	90	This study
Xyn10A	*Acidothermus cellulolyticus* 11B^T^	*Bacillota*	Endoxylanase	90	[19]
CbXyn10A	*Caldicellulosiruptor bescii* DSM 6725^T^	*Bacillota*	Endoxylanase	85	[4]
XynA	*Clostridium acetobutylicum* ATCC 824^T^	*Bacillota*	Xylanase	60	[9]
XynA	*Dictyoglomus thermophilum* RT46B.1	*Dictyoglomata*	Xylanase	85	[20]
XynA	*Geobacillus thermodenitrificans* TSAA1	*Bacillota*	Endoxylanase	70	[12]
Mros_2091	*Melioribacter roseus* DSM 23840^T^	*Ignavibacteriota*	Endoxylanase	65	[21]
Xyn10A	*Rhodothermus marinus* DSM 4252^T^	*Bacteroidota*	Endo‐1,4‐beta‐xylanase	ND^b^	[16, 17, 18]
Xyn	*Thermoanaerobacterium thermosaccharolyticum* DSM 571^T^	*Bacillota*	Xylanase	65	[15]
XynA	*Thermotoga neapolitana*	*Thermotogata*	Endo‐1,4‐beta‐xylanase	102	[22]
TmXYN10B	*Thermotoga maritima*	*Thermotogata*	Xylanase	105	[23]

^a^ The genus and species names here are as listed in CAZy and valid phylum names are added here

^b^ Not determined or not available.

**Fig. 4:**
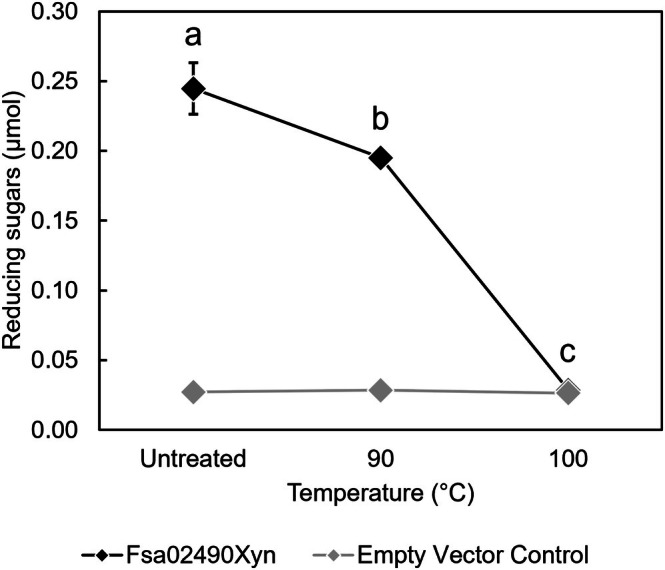
Thermostability of Fsa02490Xyn. Fsa02490Xyn was mostly stable up to 90 °C for 1 h. Incubation at 100 °C for 1 h resulted in full loss of activity on xylan from beech wood. Temperatures with a shared letter are not significantly different (*n* = 3, *P* < 0.05 via a one‐way ANOVA with *post hoc* Tukey's HSD). Fsa02490Xyn treated at 100 °C for 1 h was not significantly different from the empty vector control (*P* > 0.05 via an unpaired *t*‐test). Error bars indicate standard deviation. In some cases, error bars are smaller than symbols in the graph. Assays were done with 40.4 μg·mL^−1^ (final concentration) of Fsa02490Xyn and 0.5% substrate (final concentration) at pH 7.5 at 90 °C.

The thermostability of Fsa02490Xyn was assessed by heating the purified enzyme to 70, 80, 90, and 100 °C for 1 h and then testing for activity on xylan at 90 °C and pH 7.5 (Fig. 4). This revealed a small (20%) but significant decrease in enzyme activity after incubation at 90 °C (*P*‐value < 0.05, unpaired *t*‐test). In contrast, the enzyme lost all activity after a 1‐h incubation at 100 °C, even though the enzyme was optimally active at this temperature. The poor stability of the purified enzyme at 100 °C is consistent with the steep drop‐in activity of the enzyme between 100 °C and 120 °C (Fig. S5) and the lower optimal growth temperature of *F. sacchari*.

The kinetic parameters of Fsa02490Xyn were determined at 90 °C and a pH of 7.5 using the colorimetric substrate *p*NPX2 (Fig. S4). A Lineweaver–Burk plot was used to determine the *K*_m_, *V*_max_ and *k*_cat_/*K*_m_ values to be 2.375 mm, 1250 μm·min^−1^, and 1.259 × 10^4^ s^−1^·m ^−1^ (31.23 mL·s^−1^·mg^−1^). This *k*_cat_/*K*_m_ is relatively low compared to other GH10 xylanases (29–290 mL·s^−1^·mg^−1^) [50]. However, the relative efficiency of Fsa02490Xyn on natural substrates may be higher than on *p*NPX2, as was seen for a GH50 glucanase from *F. sacchari* that degraded natural glucans more effectively than *p*NP‐β‐D‐glucopyranoside [51]. Also, some activity on *p*NPX2 would not be detected by the assay. Specifically, *p*NPX2 consists of *para*‐nitrophenol linked to xylobiose (Xyl[β1,4]Xyl), and activity is measured by the release of *p*NP from xylobiose, causing an increase in absorbance at 400 nm. However, cleavage of the β‐1,4‐glycosidic linkage between xylose monomers would yield *p*NP‐β‐d‐xylopyranoside and xylose, neither of which would affect the absorbance at 400 nm. Thus, potential cleavage of the β‐1,4‐glycosidic linkage in xylobiose by Fsa02490Xyn is not reported by this assay.

### Evolution of Fsa02490Xyn within *Fervidibacteria* and characterized GH10 enzymes

**Fig. 5:**
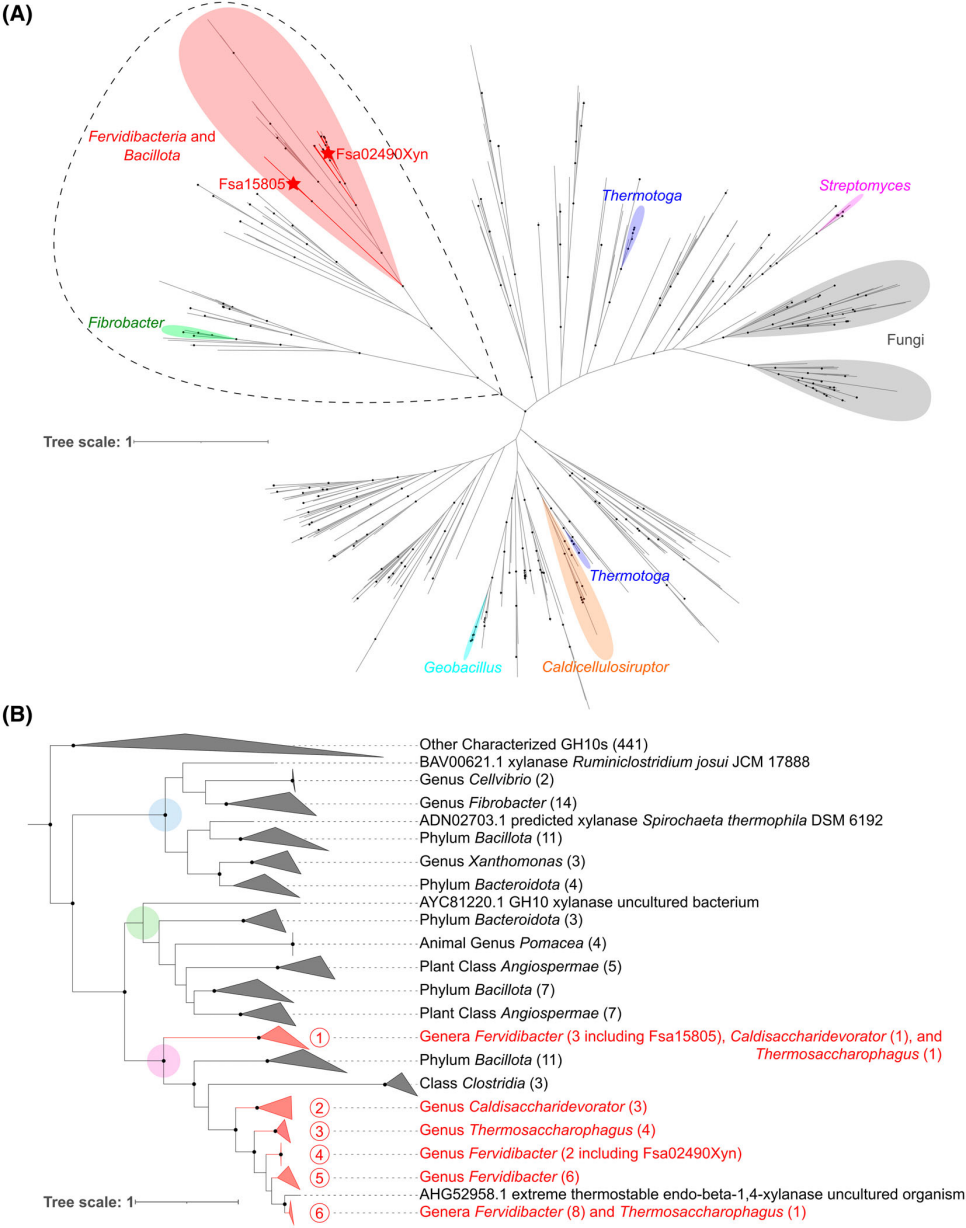
Phylogenetic analysis of GH10. (A) A maximum likelihood analysis of GH10 created using IQTree with UFBoot and SH‐aLRT branch support. Sequences from *Fervidibacteria* are indicated by red branches. Fsa02490Xyn and Fsa15805 are denoted by red stars. Clusters of 10+ sequences from the same genera are marked with colored highlights. The sequences shown in panel (B) are encircled with a black dashed line. Well‐supported nodes (UFBoot ≥ 95% and SH‐aLRT ≥ 0.9) are indicated by a black dot. The scale bar indicates the number of amino acid changes per site. (B) A subtree of the same maximum likelihood tree showing only *Fervidibacteria* GH10 enzymes and their closest relatives. Sequences are clustered into six *Fervidibacteria* clades (shown in red with the number of sequences numbered at the tips) and labeled at their lowest common taxonomic rank. Red numbers in red circles correspond to clades discussed in the text. Well‐supported nodes (UFBoot ≥ 95% and SH‐aLRT ≥ 0.9) are indicated by black dots. The scale bar indicates the number of amino acid changes per site. Members of major clades (indicated here by blue, green, and pink circles) are highlighted in Table S4.

A phylogenetic analysis was done to identify relationships between Fsa02490Xyn, all characterized GH10 enzymes from CAZy (519 total), and other putative GH10 enzymes from high‐quality *Fervidibacteria* genomes (28 total) [25] (Fig. 5). Overall, the phylogeny revealed a poor correspondence between the GH10 phylogeny and the taxonomy of the organisms, suggesting large‐scale horizontal gene transfer (HGT) across the phylogeny. HGT has previously been noted as a major force in GH evolution in both bacteria and eukaryotes [52, 53, 54], including GH10 enzymes [42]. However, the 28 GH10 enzymes from the *Fervidibacteria* genomes formed a well‐supported clade on the phylogeny along with 15 other enzymes. This clade is marked in red for clarity in Fig. 5A and shown in detail in Fig. 5B, also with enzymes from *Fervidibacteria* in red. Fourteen of the other enzymes related to *Fervidibacteria* GH10 enzymes were from diverse *Bacillota*, including moderately thermophilic xylanases from cultivated thermophiles classified as *Alicyclobacillus* [55], *Thermoanaerobacterium* [56], *Thermoclostridium* [57], and *Herbinix* [58] and xylanases expressed from fosmids obtained from solid digesta collected from cattle rumens [59] and pulp wastewater [60]. The other related GH10 enzyme, Xyn10K (AHG52958.1), was expressed from a fosmid obtained from an alkaline hot spring in Kamchatka, Russia [48]. Xyn10K was closely related to Fsa02490Xyn and other *Fervidibacteria* enzymes in the phylogeny and was optimally active on beech‐wood xylan at 95 °C. Although Xyn10K was not associated with a genome, we believe that it was likely derived from a member of the *Fervidibacteria*.

Among *Fervidibacteria* GH10 enzymes (Fig. 5B), the two *F. sacchari* PD1^T^ enzymes, Fsa02490Xyn and Fsa15805, were distantly related, and the GH10 phylogeny was poorly correlated with *Fervidibacteria* taxonomy. For example, although a few genus‐specific clades of *Fervidibacteria* GH10 enzymes were apparent (Fig. 5B, clades 2–5), clade 1 contained very similar GH10 enzymes from all three known genera of *Fervidibacteria* and clade 6 contained GH10 enzymes encoded by several *Fervidibacter* species along with a single GH10 enzyme from *Thermosaccharophagus tengchongensis* DRTY‐6.bins54, which belongs to a separate family of *Fervidibacteria*. Thus, the phylogeny suggests a complex pattern of evolution involving HGT of GH10 enzymes within distinct *Fervidibacteria* and thermophilic *Bacillota* that is likely facilitated by cohabitation in geothermal environments. The exceptionally high thermostability and optimal catalytic temperatures of *Fervidibacteria* GH10 enzymes would also facilitate selection of horizontally transferred GH10 enzymes among *Fervidibacteria*. A phylogenetic analysis involving uncharacterized GH10 enzymes, including those from *Armatimonadota*, could enable a deeper understanding of the evolution of these enzymes within *Armatimonadota* and between other organisms.

## Conclusions

The characterization of Fsa02490Xyn described here is only the second well‐characterized GH enzyme known to come from a member of the *Armatimonadota*, with the other enzyme being Fsa16295Glu, a hyperthermophilic glucanase from *F. sacchari* that established a new subfamily of GH50, GH50_3 [51]. However, we also believe Xyn10K may be derived from a member of *Fervidibacteria*, likely the genus *Fervidibacter*. The *Armatimonadota*, formerly called candidate phylum OP10, is widely distributed in nature but is resistant to laboratory cultivation [61]. Recent analysis of all available high‐quality and medium‐quality *Armatimonadota* genomes, including 65 *Fervidibacteria* genomes, showed that *Armatimonadota* genomes contain 51–349 annotated GHs representing 25–108 GH families, suggesting a broad importance for polysaccharide catabolism across the entire phylum. We suggest that additional studies of polysaccharide metabolism in *Armatimonadota* would be fruitful to better understand the biology of this group, improve culturability, and possibly lead to industrially useful enzymes.

The function of Fsa02490Xyn as an endo‐β‐1,4‐xylanase with maximum activity on xylan, shown here, is interesting within the context of both RNA‐Seq and proteomics data [25], both showing Fsa02490Xyn to be among the most highly expressed GH enzymes when *F. sacchari* was grown on eight different sole carbon sources (RNA‐Seq) or five different carbon sources (proteomics), even though xylan was not used in those experiments. This expression pattern implies a broad role for Fsa02490Xyn in polysaccharide catabolism even when its optimal substrate is not present. The other *F. sacchari* GH10 enzyme, Fsa15805, was expressed at much lower levels under all growth conditions and the heterologously expressed and purified enzyme was not active in broad screens, although negative activity results may be due to problems with folding, cofactor synthesis, maturation, or other factors. GH10 enzymes are typically less sterically hindered by side groups and have broader substrate recognition and activity than other xylanases [62]. The relatively broad substrate ranges of GH10 enzymes and their tolerance of side chains may be important to *Fervidibacteria* and other thermophiles yet the sources and structures of xylans available to them are not well understood.

## Conflict of interest

The authors declare no conflict of interest.

## Peer review

The peer review history for this article is available at https://www.webofscience.com/api/gateway/wos/peer‐review/10.1002/2211‐5463.70072.

## Author contributions

BPH, JKC, IKB, J‐FC, RM, and MH‐S conceived and designed the project. NT, JKC, AMC, and MP acquired and analyzed the data. BPH and NT wrote the paper. All authors reviewed the manuscript before submission.

## Supporting information


**Fig. S1.** Multiple sequence alignment of selected GH10 enzymes.
**Fig. S2.** alphafold 2 CSM of Fsa02490Xyn.
**Fig. S3.** TEV protease pre‐treatment has no effect on activity.
**Fig. S4.** Lineweaver‐Burk plot for Fsa02490Xyn on pNPX2.
**Fig. S5.** Temperature range and optimum of Fsa02490Xyn.

**Table S1.** Raw data used for Fig. 2.
**Table S2.** Raw data used for Fig. 3.
**Table S3.** Raw data used for Fig. 4.
**Table S4.** GH10 proteins used in the phylogenetic analysis in Fig. 5.
**Table S5.** Raw data used for Fig. S3.
**Table S6.** Raw data used for Fig. S4.
**Table S7.** Raw data used for Fig. S5.

## Data Availability

The data that support the findings of this study are available in the Supporting Information of this article.
