# Supplementary material for: Characterization of the thermophilic xylanase Fsa02490Xyn from the hyperthermophile Fervidibacter sacchari belonging to glycoside hydrolase family 10
Source: FEBS Open Bio. 2025 Aug 29;15(10):1629–42. doi: 10.1002/2211-5463.70072 (PMC12485897; doi:10.1002/2211-5463.70072)

Supplementary Information for:

Characterization of the thermophilic xylanase Fsa02490Xyn from the hyperthermophile *Fervidibacter sacchari* belonging to glycoside hydrolase family GH10

Nicole Torosian[1†], Jonathan K. Covington[1†], Allison M. Cook[1], Nancy O. Nou[1], Marike Palmer[1,2], Ritesh Mewalal[3], Miranda Harmon-Smith[3], Ian K. Blaby[3], Jan-Fang Cheng[3], Matthias Hess[4], Brian P. Hedlund[1,5*]

[1]School of Life Sciences, University of Nevada, Las Vegas, Las Vegas, NV, USA
[2]Department of Microbiology, University of Manitoba, Winnipeg, MB, Canada
[3]US Department of Energy Joint Genome Institute, Lawrence Berkeley National Laboratory, Berkeley, CA, USA
[4]Department of Animal Science, College of Agricultural and Environmental Sciences, University of California, Davis, Davis, CA, USA
[5]Nevada Institute of Personalized Medicine, University of Nevada, Las Vegas, Las Vegas, NV, USA

Supplementary Figure 1. Multiple sequence alignment of selected GH10 enzymes. A multiple sequence alignment of five GH10s was generated using MAFFT-DASH, and visualized in Jalview v2.11.4.1. Red indicates two conserved glutamic acid residues, Fsa02490Xyn Glu201 and Glu305 (Glu166 and Glu270 in the mature form). Purple shading indicates the percent identity of a given residue. Reference sequences are detailed in Supplementary Table 1 and are as follows: AEA30147.1, *Cellulomonas fimi* ATCC 484 Xyn10A; AAD35164.1, *Thermotoga maritima* MSB8 Xyl10B; ACM59335.1, *Caldicellulosiruptor bescii* DSM 6725 CbXyn10A; AGD81833.1, *Geobacillus stearothermophilus* 1A05583 XynA. The 58 C-terminal amino acids comprising the β-sandwich-like structure in Fsa02490Xyn begin at position 387. The annotated CBM2 domain in the *Caldicellulosiruptor bescii* DSM 6725 CbXyn10A begins approximately at position 385 after a predicted intrinsically disordered proline-rich region.

```
Fsa02490Xyn/1-444     1 MKRRDFVATLL-----------------------GTTVAGAIG--YLGT  24
AEA30147.1/1-485      1 MPR--------------------------------TTPA----PGHPAR  13
AAD35164.1/1-347      1 MKI--------------------------------LPSV-LI-------   9
ACM59335.1/1-697      1 MKKRKFKILYLFLIIVLSVSFIISIVFPSFFKAAQTTST-NIN--FEGR  46
AGD81833.1/1-331      1 M------------------------------------------------   1

Fsa02490Xyn/1-444    25 ---------RGRWSVDEAIAQEEQ-------------------------  39
AEA30147.1/1-485     14 ---------GARTALRTTLAAAAA-------------------------  28
AAD35164.1/1-347        -------------------------------------------------
ACM59335.1/1-697     47 DKLTFFAYGKAKITIDQNIAQEGKKSIKVTDRKSVWDSFGIDVKDVLQR  95
AGD81833.1/1-331        -------------------------------------------------

Fsa02490Xyn/1-444       -------------------------------------------------
AEA30147.1/1-485        -------------------------------------------------
AAD35164.1/1-347        -------------------------------------------------
ACM59335.1/1-697     96 GKTWVVSAYVKHKGKKPIEFSITAIYNDGRGLKYLQLGEKIVIPNKWDK 144
AGD81833.1/1-331        -------------------------------------------------

Fsa02490Xyn/1-444    40 --------------VLSGVKERIEQHRK---------GEVTLVVQAL--  63
AEA30147.1/1-485     29 -------------TLVVGA-------------------TVVLPAQA---  42
AAD35164.1/1-347     10 --------------LLLGC-------------------VPVFSSQ----  21
ACM59335.1/1-697    145 IVAKWKPTLKNPMDLIIAIHPTVDKTTAYNVDNIQIMTEEVYQSQAVVF 193
AGD81833.1/1-331        -------------------------------------------------

Fsa02490Xyn/1-444    64 ----------------------------NGK-----------------P  67
AEA30147.1/1-485        -------------------------------------------------
AAD35164.1/1-347        -------------------------------------------------
ACM59335.1/1-697    194 KDTFESNLTNWQPRGDTVKLKIDNTKSHNGNKSLYVSGRSAFWHGVQIP 242
AGD81833.1/1-331        -------------------------------------------------

Fsa02490Xyn/1-444    68 I------------------------------------------------  68
AEA30147.1/1-485        -------------------------------------------------
AAD35164.1/1-347        -------------------------------------------------
ACM59335.1/1-697    243 VTKYLVAGKVYKFSVWLYHQSIDKQGFGLTIQRKMANDEQYKYDWITGS 291
AGD81833.1/1-331        -------------------------------------------------

Fsa02490Xyn/1-444       -------------------------------------------------
AEA30147.1/1-485        -------------------------------------------------
AAD35164.1/1-347        -------------------------------------------------
ACM59335.1/1-697    292 QIEGDGWVEISGNYYVPKDGKIEELVFCVSSWNPTLAFWVDDVTISDPF 340
AGD81833.1/1-331        -------------------------------------------------

Fsa02490Xyn/1-444    69 ----PNAELTLTQTRH--EFLFG-CNIFRWGRIPDPKR-EELYRERFAS 109
AEA30147.1/1-485     43 -----ATTLKEAADGAGRDF--G-FALDP-NRLSEAQY-KAIADSEFNL  81
AAD35164.1/1-347     22 -----NVSLRELAEKL--NIYIGFAAINNFWSLSDAEKYMEVARREFNI  63
ACM59335.1/1-697    341 KLQGPNYNLPSLKEKYKEDFKVG-VAIGYGELISDID--TQFIKKHFNS 386
AGD81833.1/1-331      2 -----CSSIPSLRGVFANDFRIG-AAVNP--VTLEAQ--QSLLIRHVNS  40
```

```
Fsa02490Xyn/1-444 110 IFNYATLPFYWAGYEWERGKPNHEYIDRVVDWCQQHGITCKGHPLVWDH 158
AEA30147.1/1-485   82 VVAENAMK--WDATEPSQNSFSFGAGDRVASYAADTGKELYGHTLVWHS 128
AAD35164.1/1-347   64 LTPENQMK--WDTIHPERDRYNFTPAEKHVEFAEENDMIVHGHTLVWHN 110
ACM59335.1/1-697  387 ITPGNEMK--PESVLKGPNNYDFTIADAFVDFATKNKMGIRGHTLVWHN 433
AGD81833.1/1-331   41 LTAENHMK--FEHLQPEEGRFTFDIADQIIDFARSHHMAVRGHTLVWHN  87

Fsa02490Xyn/1-444 159 QASSPDRWLPDDFA-------EIEKLSTARVREIVQRFAGRIDIWDVVN 200
AEA30147.1/1-485  129 Q--LPD-WA-KNLNGS-----AFESAMVNHVTKVADHFEGKVASWDVVN 168
AAD35164.1/1-347  111 Q--LPG-WI----TGREWTKEELLNVLEDHIKTVVSHFKGRVKIWDVVN 152
ACM59335.1/1-697  434 Q--TPD-WFFKDENGNFLKKDELLKRLKNHIYTVVSRYKGKIYAWDVVN 479
AGD81833.1/1-331   88 Q--TPS-WVFQDSQGHFVGRDVLLERMKSHISTVVQRYKGKVYCWDVVN 133

Fsa02490Xyn/1-444 201 EPTDIWRFP-TKMSRWARQIGNVPYTILHLKVAREANPKATLLVNDYR- 247
AEA30147.1/1-485  169 EAFADGGR-RQDSAFQQKLGN-GYIETAFRAARAADPTAKLCINDYNV 215
AAD35164.1/1-347  153 EAVSDSGT--YRESVWYKTIGP-EYIEKAFRWAKEADPDAILIYNDYSI 198
ACM59335.1/1-697  480 EAIDETQPDGYRRSNWYNICGP-EYIEKAFIWAHEADPQAKLFYNDYN- 526
AGD81833.1/1-331  134 EAVADEGSEWLRSSTWRQIIGD-DFIQQAFLYAHEADPEALLFYNDHN- 180

Fsa02490Xyn/1-444 248 -----TDDAYFKVLQQLKDEKGQWLFDAVGIQSHMHGGIWSPQRTWQVC 291
AEA30147.1/1-485  216 EGINAKSNSLYDLVKDFK-ARGVPL-DCVGFQSHLIVGQ-VPGDFRQNL 261
AAD35164.1/1-347  199 EEINAKSNFVYNMIKELK-EKGVPV-DGIGFQMHIDYRGLNYDSFRRNL 245
ACM59335.1/1-697  527 TEIPQKRMFIYNMIKNLK-AKGVPI-HGIGLQCHINIDNPSVEDIEETI 573
AGD81833.1/1-331  181 ECFPEKREKIYTLVKSLR-DKGIPI-HGIGMQAHWSLNRPTLDEIRAAI 227

Fsa02490Xyn/1-444 292 ERFAQL-GLPLHFTETTIVSGPRVDRER-WGETTPEGEERQAEETVRFY 338
AEA30147.1/1-485  262 QRFADL-GVDVRITELDIRMRTPSDATK---------LATQAADYKKVV 300
AAD35164.1/1-347  246 ERFAKL-GLQIYITEMDVRIPLSGSEEY--------YLKKQAEVCAKIF 285
ACM59335.1/1-697  574 KLFSTIPGLEIQITELDMSFYQWGSSVY-YAEPSREMLLKQAKKYYELF 621
AGD81833.1/1-331  228 ERYASL-GVILHITELDISMFEFDDHRKDLAAPTDEMVERQAERYEQIF 275

Fsa02490Xyn/1-444 339 TLLFSHPAV-QAITWWDFSDDGAWMGA--------PAGWLRRDMSPKPV 378
AEA30147.1/1-485  301 QACMQVTRC-QGVTVWGITDKYSWVPD--VFPGEGAALVWDASYAKKPA 346
AAD35164.1/1-347  286 DICLDNPAV-KAIQFWGFTDKYSWVPG--FFKGYGKALLFDENYNPKPC 331
ACM59335.1/1-697  622 NLFKKYKNVIKSVTFWGLKDDNSWLRG--VFNKPDFPLLFDEHYDGKPA 668
AGD81833.1/1-331  276 SLFKEYRDVIQNVTFWGIADDHTWLDHFPVQGRKNWPLLFDEQHKPKPA 324

Fsa02490Xyn/1-444 379 YERMLELI----------------------------------KGEWWTK 393
AEA30147.1/1-485  347 YAAVMEAFGASPTPTPTTPTPTPTTPTPTPTSGPAGCQVLWGVNQWNTG 395
AAD35164.1/1-347  332 YYAIKEVL----------------------------------------- 339
ACM59335.1/1-697  669 FWALIDYS--------ILPQ-NANLPTP-----PA-------------- 689
AGD81833.1/1-331  325 FWRVVNI------------------------------------------ 331

Fsa02490Xyn/1-444 394 ASGKTDSKGVWRTRAYYGDYELTIRTPDGRVSKQKLS------------ 430
AEA30147.1/1-485  396 FTANVTVKNT--SSAPVDGWTLTFSFPSGQQVTQAWSSTVTQSGSAVTV 442
AAD35164.1/1-347  340 -EKKIEERK---------------------------------------- 347
ACM59335.1/1-697  690 -IPKVKAKK---------------------------------------- 697
AGD81833.1/1-331      -------------------------------------------------
```

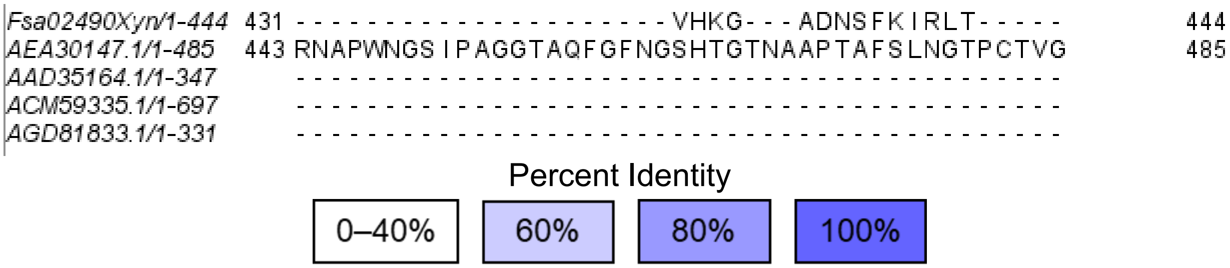

Fsa02490Xyn/1-444 431 ---------------------VHKG---ADNSFKIRLT----- 444
AEA30147.1/1-485 443 RNAPWNGSIPAGGTAQFGFNGSHTGTNAAPTAFSLNGTPCTVG 485
AAD35164.1/1-347 ---
ACM59335.1/1-697 ---
AGD81833.1/1-331 ---
Percent Identity
0–40%
60%
80%
100%

Supplementary Figure 2. Alphafold 2 CSM of Fsa02490Xyn. (A) CSM of the native, mature Fsa02490Xyn monomer with conserved glutamic acids Glu166 (green) and Glu270 (red) shown. The N- (N) and C (C)-termini are labeled. Viewed from the side showing the "salad bowl" structure typical of (β/α)8 TIM barrels. Below the predicted (β/α)8 TIM barrel is a predicted C-terminal β-sandwich-like structure similar to some CBMs but composed of only seven β-sheets and one pair of juxtaposed β-sheets being parallel instead of antiparallel. This structure was not annotated using HMMER, dbCAN-sub, DIAMOND, or a separate analysis using InterPro scan. (B) PAE plot of the native, mature Fsa02490Xyn indicating low distance error between each residue pair. (C) Top-down view of the (β/α)8 TIM barrel of the native, mature Fsa02490Xyn with C-terminal ends of the β-barrel containing putative catalytic glutamic acid residues pointed up. Conserved glutamic acid residues shown. (D) CSM of the non-native Fsa024Xyn (blue) from the side showing the typical "salad bowl" structure and the C-terminal β-sandwich-like structure, with an N-terminal 6 x His tag (green), GB1 solubility tag (yellow), and TEV protease site (purple). The N- (N) and C (C)-termini are labeled.

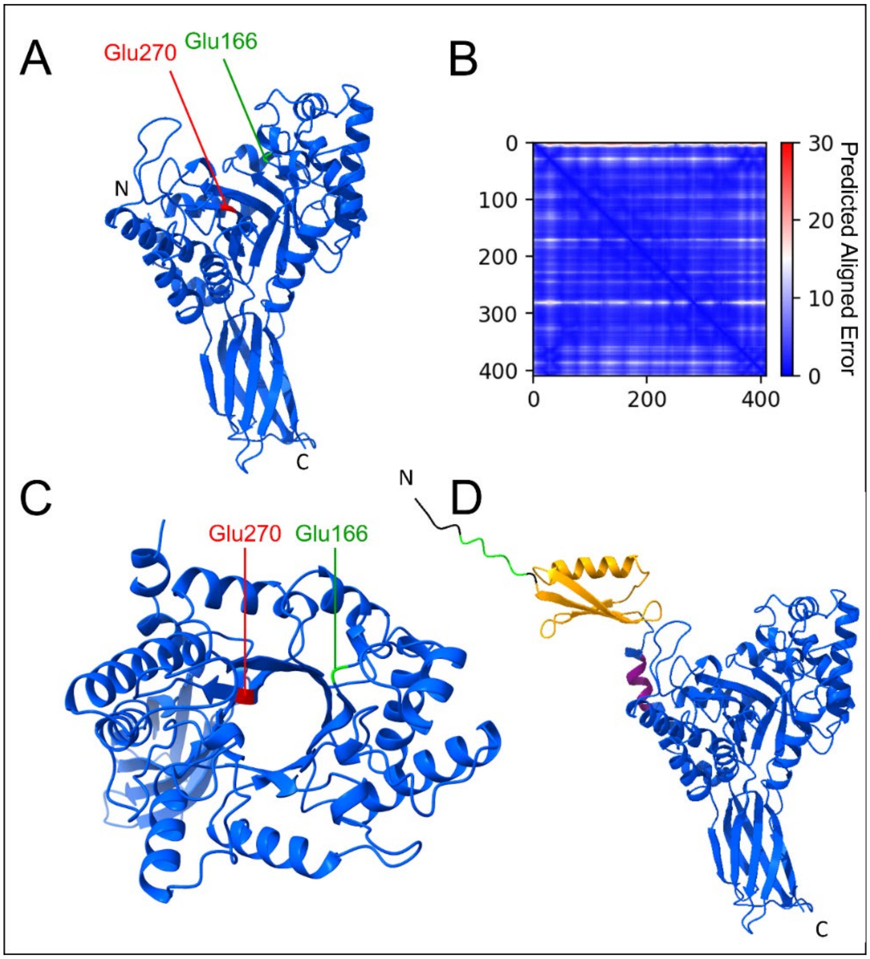

Supplementary Figure 3. TEV protease pre-treatment has no effect on activity. Fsa024Xyn was treated with TEV protease to remove the 6 x His and GB1 solubility tags to yield the native, mature Fsa02490Xyn, which did not change the activity of Fsa02490Xyn on β-glucan from oat (n = 3, $p > 0.05$ via a one-way unpaired t-test). Error bars indicate standard deviation. Assays were done with 40.4 µg/mL (final concentration) of Fsa02490Xyn and 0. 5% substrate (final concentration) at 90 ºC at pH 7.5 overnight.

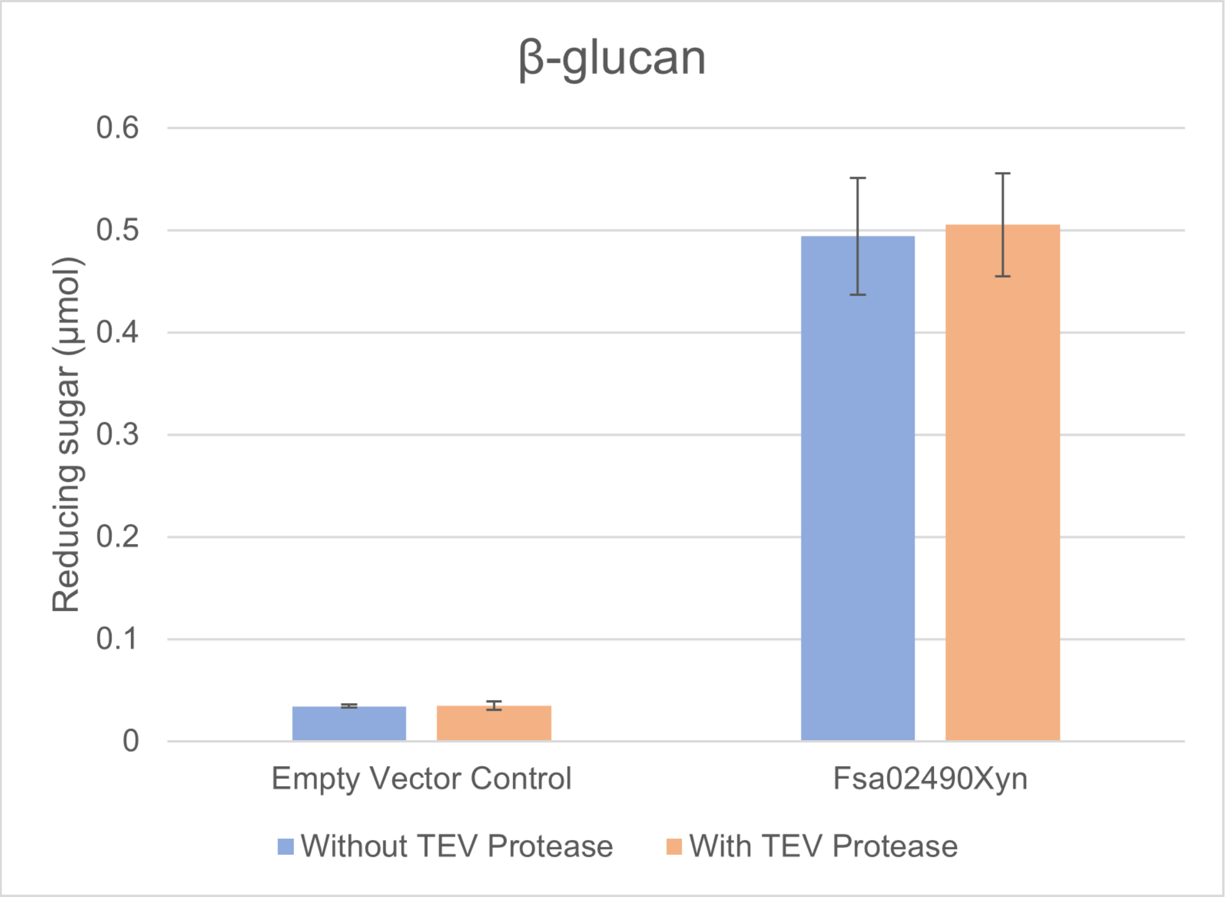

Supplementary Figure 4. Lineweaver-Burk plot for Fsa02490Xyn on pNPX2. Fsa02490Xyn was incubated with pNPX2 for 11 minutes with a range of concentrations (n = 3; 0.0626, 1.25, 2.5, 10, and 20 mM), resulting in a linear Lineweaver-Burk plot (R2 = 0.9795). The x- and y-intercepts used to calculate Km and Vmax values of 2.375 mM and 1250 µM/min. Assay done at 90 ºC and pH 7.5.

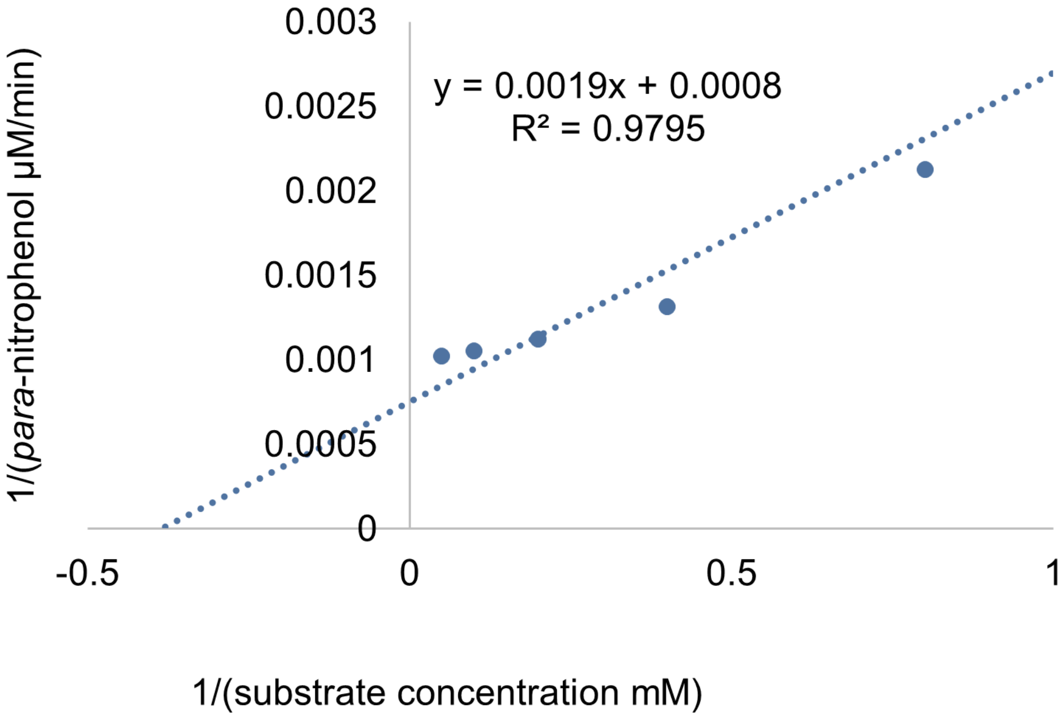

Supplementary Figure 5. Temperature range and optimum of Fsa02490Xyn. Fsa02490Xyn was optimally active at 90–100 °C. Treatments with a shared letter are not significantly different (n = 3, $p < 0.05$ via a one-way ANOVA with *post-hoc* Tukey's HSD). Error bars indicate standard deviation. Fsa02490Xyn was active at all temperatures tested when compared to the empty vector control ($p < 0.05$ via unpaired *t*-tests). Assays were done with 40.4 µg/mL (final concentration) of Fsa02490Xyn and 0.5% substrate (final concentration) at pH 7.5. The exact same data set was previously published in Reference 25.

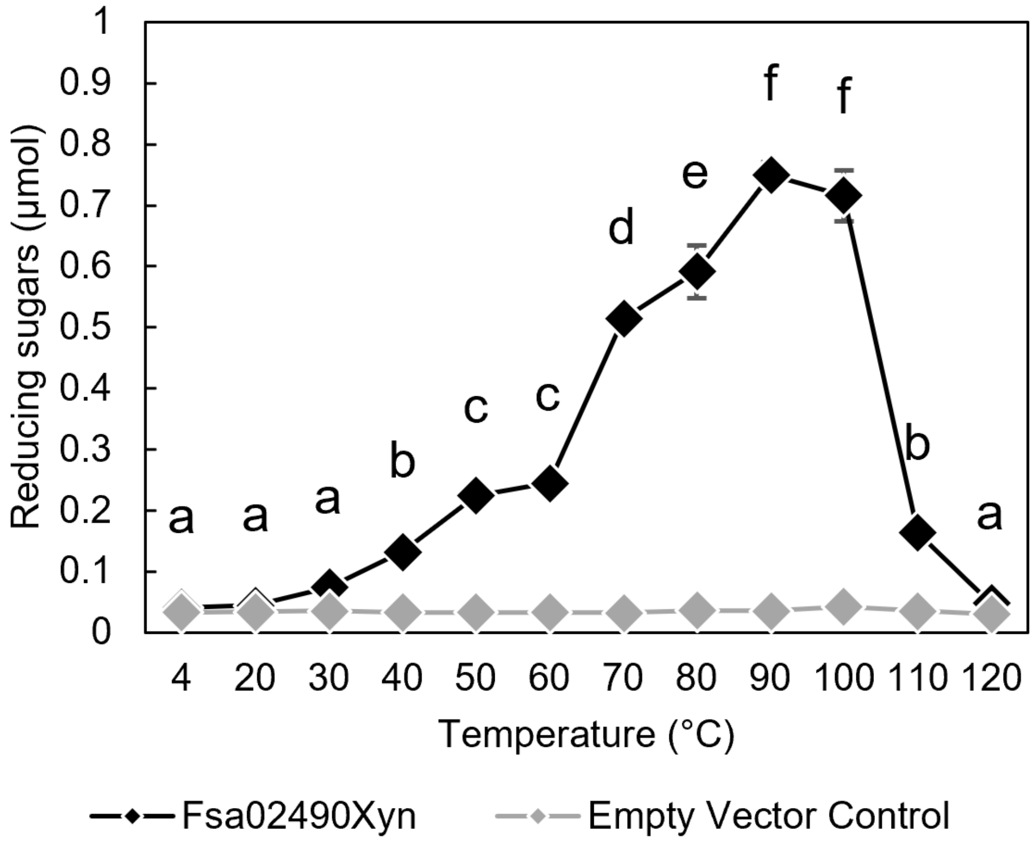

Supplement: Supplementary file 1 — Fig. S1. Multiple sequence alignment of selected GH10 enzymes. Fig. S2. alphafold 2 CSM of Fsa02490Xyn. Fig. S3. TEV protease pre‐treatment has no effect on activity. Fig. S4. Lineweaver‐Burk plot for Fsa02490Xyn on pNPX2. Fig. S5. Temperature range and optimum of Fsa02490Xyn. [file FEB4-15-1629-s001.pdf]
